# Identification of two novel *PRPF31* mutations in Chinese families with non‐syndromic autosomal dominant retinitis pigmentosa

**DOI:** 10.1002/mgg3.1537

**Published:** 2020-10-21

**Authors:** Li Cao, Chunyan Peng, Jing Yu, Wei Jiang, Jiyun Yang

**Affiliations:** ^1^ College of Medical Technology Chengdu University of Traditional Chinese Medicine Chengdu PR China; ^2^ The Key Laboratory for Human Disease Gene Study of Sichuan Province Prenatal Diagnosis Center Sichuan Provincial People's Hospital the University of Electronic Science and Technology of China Chengdu PR China; ^3^ School of Medicine University of Electronic Science and Technology of China Chengdu PR China

**Keywords:** *PRPF31*, retinitis pigmentosa, variation, whole exome sequencing

## Abstract

**Background:**

Retinitis pigmentosa is a heterogeneous group of inherited retinal diseases leading to progressive vision loss. It has been estimated that the etiology is still unclear in 22%‐40% of cases, indicating that many novel pathogenic variations related to RP remain unidentified in many patients. In this study, our aim was to investigate the disease‐causing variants and function of the variants in two Chinese families with non‐syndromic autosomal dominant retinitis pigmentosa (adRP).

**Methods:**

Clinical data and peripheral blood DNA samples were collected. Whole exome sequencing (WES) was conducted to screen for variations. Then, the expression of green fluorescent protein (GFP)‐fused wild‐type PRPF31 protein and its variants was evaluated via western blotting and GFP fluorescence detection *in vitro*.

**Results:**

Two novel heterozygous variants of *PRPF31* (NM_015629.4): c.855+5G>A and c.849_855del (p.Pro284Ilefs*35) were identified respectively in two families. The variant c.855+5G>A is co‐segregated with the disease in adRP‐01 family. The pedigree analysis result for c.849_855del (p. Pro284Ilefs*35) shows an inheritance pattern with incomplete penetrance for adRP‐02 family. The RT‐PCR analysis shows the *PRPF31* gene c.855+5G>A leading to the missing from the 997th to the 1405th positions of the *PRPF31* gene (NM_015629.4) cDNA. The expressions of the mutant GFP‐fused PRPF31 protein were not detected in HEK293 cells or Cos7 cells via western blotting and immunofluorescence.

**Conclusions:**

Our findings identified two novel variants in *PRPF31* in two Chinese families with adRP, expanding the mutational spectrum of this gene. Functional analysis reveals that these variants lead to the truncation of the PRPF31 protein.

## Graphical abstract

In this work, we reported two novel heterozygous variants of PRPF31, c.855+5G>A and c.849_855delGCCACCG (p.Pro284Ilefs*35), in two Chinese families with autosomal dominant RP (adRP). These variants of PRPF31 have never been previously reported. Functional analysis revealed that these variants lead to the truncation of the PRPF31 protein.

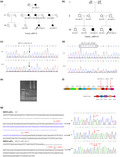

## 1 INTRODUCTION

Retinitis pigmentosa (RP, OMIM＃268000) is a heterogeneous group of inherited retinal diseases leading to progressive vision loss (Al‐Merjan et al., 2005; Hu, 1987; Xu et al., 2006). RP is a leading cause of visual disability, affecting one in 2500‐7000 people (Parmeggiani, 2011). Patients with RP usually begin experiencing night blindness before the onset of visual impairment and, in some cases, the eventual development of irreversible vision loss. The dysfunction and death of retinal photoreceptors are the most common causes of RP (Zhang et al., 2016). It is a form of retinal malnutrition (Hartong et al., 2006; Rosenberg, 2003). RP can be either non‐syndromic or syndromic. Non‐syndromic RP refers to lesions involving only the eyes, while syndromic RP refers to lesions involving multiple organs, which can lead to multifunctional disorders. Non‐syndromic RP can be inherited through multiple modes: autosomal dominant RP (adRP), autosomal recessive RP (arRP), or X‐linked RP (xlRP). Rare digenic forms can also occur. Digenic RP is observed in individuals who are double heterozygotes for ROM1 and PRPH2 (Ferrari et al., 2011).Currently, at least 79 disease‐causing genes and eight loci have been reported in OMIM (www.omim.org). It has been estimated that the etiology is still unclear in 22%‐40% of cases, indicating that many novel pathogenic variations related to RP remain unidentified (Salmaninejad et al., 2019).

The pre‐mRNA processing factor 31 (*PRPF31*, OMIM＃606419) gene spans approximately 18 kb of genomic DNA on 19q13.4, which encodes four different transcripts. *PRPF31* (NM_015629.4) is the most widely expressed transcript, which consists of 14 exons and produces a protein of 499 amino acids (Martin‐Merida et al., 2017). PRPF31 is a component of a ribonucleoprotein complex known as the spliceosome, which catalyzes the removal of introns from nuclear pre‐mRNA to produce mature mRNA molecules (Zhou et al., 2002). PRPF31 is essential to the activation of the spliceosome (Liu et al., 2007) and is prone to the second most common genetic defect of adRP, accounting for 6%‐11% of adRP cases in different populations (Coussa et al., 2015; Sullivan et al., 2013; Xu et al., 2012). More than 196 disease‐causing variants have been reported in *PRPF31*.

Here, we reported two novel heterozygous variants of *PRPF31*, c.855+5G>A, and c.849_855del (p. Pro284Ilefs*35), in two Chinese families with adRP. These variants of *PRPF31* have never been previously reported. Functional analysis revealed that these variants lead to the truncation of the PRPF31 protein.

## 2 MATERIALS AND METHODS

### 2.1 Ethical compliance

All procedures performed in this study involving human participants were in accordance with the Helsinki declaration. All participants signed for written informed consent. This study was approved by the Institutional Review Boards of Sichuan Provincial People's Hospital.

### 2.2 Subjects

**FIGURE 1:**
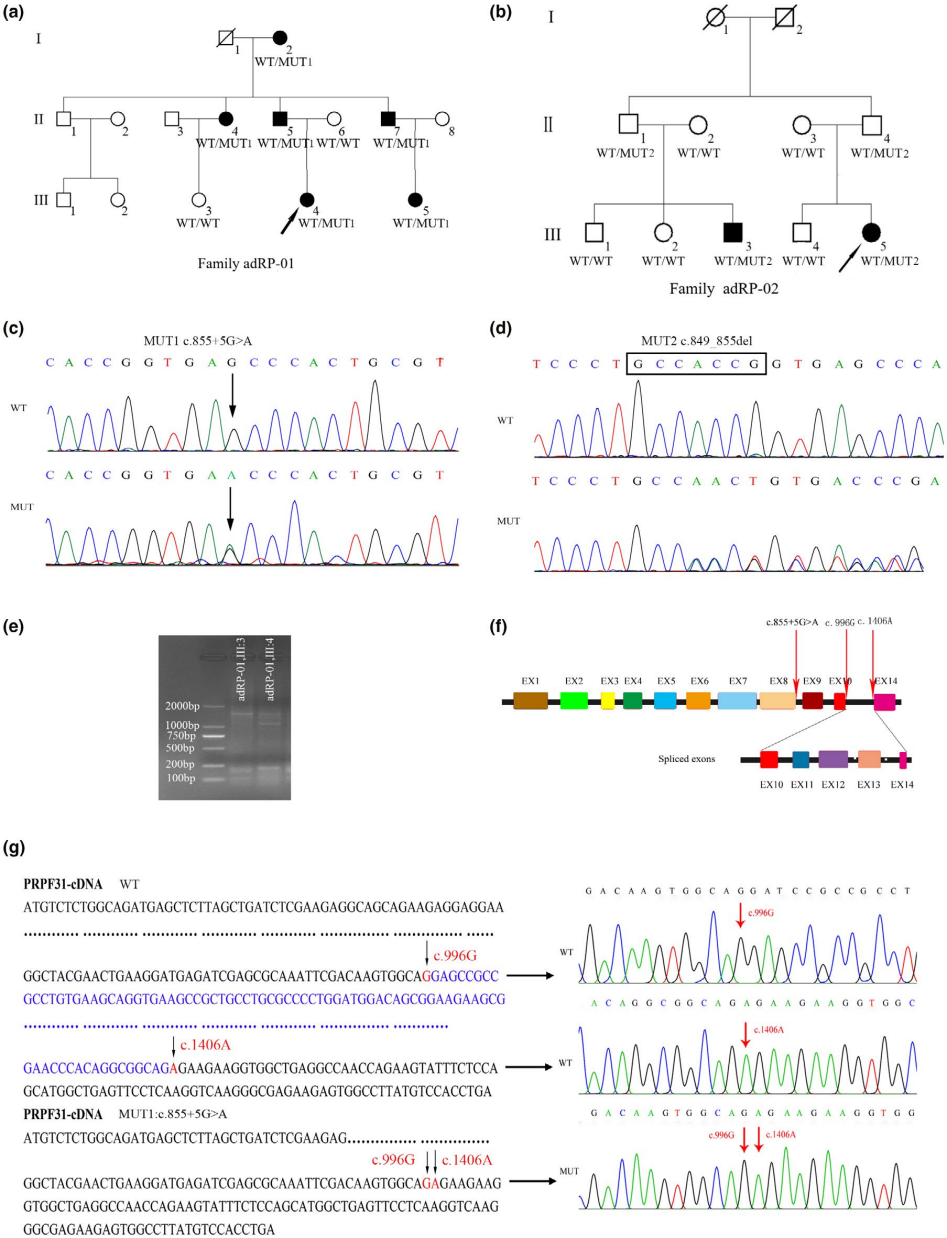
Pedigrees of two families with adRP and *PRPF31* (NM_015629.4) variations were identified by Sanger sequencing in participants. (a) Pedigree of adRP‐01 family. WT/MUT1 represents the proband and affected individual carrying heterozygous variant c.855+5G>A. WT/WT indicate wild‐type. Results shows that the novel c.855+5G>A variant in *PRPF31* shows complete co‐segregation with the disease phenotype in adRP‐01 family. (b) Pedigree of adRP‐02 family. WT/MUT2 represents the proband and individual carrying heterozygous variant c.849_855del (p.Pro284Ilefs*35). WT/WT indicates wild‐type. These results show the inheritance pattern is autosomal dominant inheritance disease with incomplete penetrance in adRP‐02 family. (c) Diagram of sanger sequencing of variant (MUT1): c.855+5G>A. (d) Diagram of sanger sequencing of variant (MUT2):c.849_855del (p.Pro284Ilefs*35). (e) Electrophoretogram of RT‐PCR for the proband and the unaffected member. Two PCR fragment of 1.5 and 1.1 kb were observed the proband (adRP‐01 III:4) and a 1.5 kb fragment were obtained from the unaffected member (adRP‐01 III:3). (f) Diagram of sanger sequencing for RT‐PCR production from the proband (adRP‐01III:4) and the unaffected member. The results show that the 1.1‐kb fragments from the proband showed deletion from the 997th to 1405 positions of *PRPF31* gene (NM_015629.4) cDNA. (g)Schematic diagram of alternative splicing isoforms. The novel c.855+5G>A variant results in the deletion of the 409 nucleotides from exon 10 to exon 14.

Two autosomal dominant RP (adRP) pedigrees were recruited. Peripheral blood samples from the eight participants of the pedigree adRP‐01 were obtained, including six RP patients (Figure 1a). The pedigree adRP‐02 consisted of nine members (Figure 1b), including two affected members. Ophthalmological examinations were performed on participants from two families, including visual acuity, fundus examination, and electroretinography (ERG).

### 2.3 DNA and RNA extraction

Peripheral blood of all participants from the two adRP families was collected in EDTA anticoagulant tubes, and then, genomic DNA was isolated by using the TIANGEN Blood DNA Kit (TIANGEN, Beijing, China) according to the manufacturer's protocol. The RNA was extracted using TRIzol (TransGen, Beijing, China), then the total RNA (100 ng) of each sample was reverse‐transcribed to cDNA using the TransScript Reverse Transcription System (TransGen). DNA and cDNA integrity was detected using 1% of agarose gel electrophoresis.

### 2.4 Whole exome sequencing and mutation analysis

Whole exome sequencing was performed on the genomic DNA samples of the probands (adRP‐01,III:4 and adRP‐02,III:5) of the two adRP families by MyGenostics Technology, Inc. Sequencing data analysis and annotation of variants were carried out as previously described(Liu et al., 2016). Variants were categorized as either benign, likely benign uncertain significance, likely pathogenic, or pathogenic variants, according to the interpretation guidelines of the ACMG (Richards et al., 2015).

### 2.5 Validation of variants and co‐segregation analysis

Candidate variants and segregation were validated using Sanger sequencing. The genomic regions containing the variants were amplified. PCR products were sequenced using an ABI 3730XL Genetic Analyzer (Applied Biosystems, Foster City, CA) according to manuals for the BigDye^TM^ Terminator v3.1 Cycle Sequencing Kits. Primers were designed using Primer3 (http://primer3.ut.ee/). Primers (c.855+5G>A‐F, c.855+5G>A‐R; c.849_855del ‐F, c.849_855del ‐R) are listed in Table S1.

### 2.6 In silico analysis

Conservation analysis of the protein sequences was performed via multiple amino acid sequence alignment, from different species, using HomoloGene (https://www.ncbi.nlm.nih.gov/homologene). To explore the effects of the *PRPF31* variant on protein structure, the online protein model prediction server SWISS‐MODEL (https://www.swissmodel.expasy.org/) was used to predict the protein structure of *PRPF31* variants.

### 2.7 Reverse transcriptase PCR

Total RNA was extracted from the nucleated cells of the peripheral blood taken from the proband and an unaffected individual of family adRP‐01. *PRPF31* (NM_015629.4) cDNA was synthesized and RT‐PCR was performed for the detection of *PRPF31* mRNA. Primers (*PRPF31*‐cDNA‐F; *PRPF31*‐cDNA‐R) for RT‐PCR and sequencing of cDNA are listed in Table S1.

### 2.8 Construction of plasmids

*PRPF31* (NM_015629.4) cDNA was cloned into *pEGFP*‐*N1* using NEBuilder^®^ HiFi DNA Assembly Cloning Kit (New England Biolabs, Ipswich, MA) according to the relevant manuals, namely *pEGFP*‐*N1*‐*PRPF31*. The primers used to amplify the *pEGFP*‐*N1* linear plasmid (pEGFP‐N1‐F; pEGFP‐N1‐R) are listed in Table S1. Two mutations (c.855+5G> A: missing from the 997th to 1405th positions of *PRPF31* cDNA, c.849_855del) were introduced into the *pEGFP*‐*N1*‐*PRPF31* using a Q5^®^ Site‐Directed Mutagenesis Kit (New England Biolabs) according to the relevant manuals, namely *pEGFP*‐*N1*‐*PRPF31M1* and *pEGFP*‐*N1*‐*PRPF31M2* (Table S1). The constructed vectors were confirmed via Sanger sequencing.

### 2.9 Cell culture, transfection, and western blotting

HEK293 and Cos7 cells were cultured in Dulbecco's modified Eagle's medium (DMEM; Gibco, Grand Island, NY), which contained 10% fetal bovine serum (FBS; Gibco), 100 unit/mL penicillin, and 100 μg/mL streptomycin. HEK293 cells were transfected with *pEGFP*‐*N1*‐*PRPF31* plasmid and mutant plasmids according to the manufacturer's instructions for the operation of Lipofectamine 3000 (Invitrogen). After 36 hours of transfection, proteins were extracted from the cells using RIPA cell lysate (BOSTER) with a protease inhibitor cocktail (Bimake). Twenty micrograms of protein were separated by SDS‐PAGE, transferred onto a PVDF membrane, which was incubated overnight at 4℃ with anti‐GFP antibodies (Proteintech), and then, incubated with goat anti‐rabbit secondary antibodies (Proteintech). GAPDH was used as an internal control. Bands were analyzed using a gel documentation system (Bio‐Rad Laboratories).

### 2.10 Immunocytochemistry

Cos7 cells were seeded in 24‐well plates on poly‐L‐lysine‐coated glass coverslips and transfected with 1 μg of each plasmid (including the WT plasmid and mutant plasmids). Cells were fixed for 15 minutes using 4% of paraformaldehyde in PBS at 36 hours post transfection. For blocking the nonspecific antibody binding sites and increasing cell permeability, the cells were incubated in PBS containing 5% of normal donkey serum (NDS; Solarbio, Beijing, China) and 0.3% Triton X‐100 for 2 hours. Subsequently, cells were incubated for 2 hours with 4′,6‐diamidino‐2‐phenylindole (DAPI). GFP fluorescence was detected directly using a confocal microscope (Zeiss LSM 800). All experiments were repeated three times.

## 3 RESULTS

### 3.1 Clinical assessments

In the pedigree adRP‐01, eight individuals consented to participate in the project, including six RP patients and two unaffected individuals (Figure 1a). The proband (III:4) of adRP‐01 was a 26‐year‐old female who presented symptoms of night blindness during early childhood. The fundus examination presented a clear border of binocular papillae and a large bone‐like pigmentation in the peripheral part of the retina. A reduction of ERG in OD and normal ERG in OS were observed in the proband. A B‐type ultrasound scan showed vitreous opacity of both eyes and posterior vitreous detachment of the right eye. OCT images showed macular holes in the left eye. Five other patients from this family were diagnosed with RP by two ophthalmologists. These patients presented typical RP symptoms, including night blindness, bone‐like pigmentation in the peripheral part of the retina, and peripheral vision loss.

In the pedigree adRP‐02, two members, a male and a female, were diagnosed with RP by two ophthalmologists. No clinical symptoms of RP were observed in the other seven subjects (Figure 1b). The clinical data of the members from the two adRP families are listed in Table 1.

**TABLE 1:** Clinical characteristics of two adRP families.

Family	SubjectID	Age/sex	Phenotype	Age of onset	Age at exam	Visual acuity (OD/OS)	Genotype
adRP−01	I:2	83/F	RP	About 50	‐	OD:0.7 OS:0.6	WT/MUT1^a^
	II:4	56/F	RP	Early childhood	52	OD:0.7 OS:0.7	WT/MUT1
	II:5	52/M	RP	Early childhood	48	OD:0.6 OS:0.6	WT/MUT1
	II:6	51/F	Unaffected	—	—	OD:0.8 OS:0.7	WT/WT
	II:7	49/M	RP	Early childhood	45	OD:0.6 OS:0.6	WT/MUT1
	III:3	21/F	Unaffected	—	—	OD:1.0 OS:0.9	WT/WT
	III:4	26/F	RP	About 8	22	OD:0.6 OS:0.1	WT/MUT1
	III:5	20/F	RP	Early childhood	16	OD: 0.8 OS:0.7	WT/MUT1
adRP−02	II:1	72/M	Unaffected	—	—	OD:0.7 OS:0.7	WT/MUT2^b^
	II:2	70/F	Unaffected	—	—	OD:0.7 OS:0.7	WT/WT
	II:3	63/F	Unaffected	—	—	OD:0.8 OS:0.8	WT/WT
	II:4	70/M	Unaffected	—	—	OD:0.8 OS:0.7	WT/MUT2
	III:1	49/M	Unaffected	—	—	OD:0.9 OS:0.9	WT/WT
	III:2	46/F	Unaffected	—	—	OD: 0.9 OS:1.0	WT/WT
	III:3	46/M	Affected	Early childhood	44	OD: 0.7 OS:0.7	WT/MUT2
	III:4	40/M	Unaffected	—	—	OD:0.9 OS:0.8	WT/WT
	III:5	28/F	Affected	About 14	26	OD:0.7 OS:0.6	WT/MUT2

Abbreviations: F, female; M, male; MUT, mutation; WT, wild type.

^a^ MUT1, *PRPF31*: c.855+5G>A.

^b^ MUT2, *PRPF31*: c.849_855del; Genbank reference sequence of *PRPF31* is NM_015629.4.

### 3.2 Genetic findings

In the adRP‐01 family, WES showed that the proband (III:4) had a novel c.855+5G>A variant in *PRPF31*. This variant was not found in dbSNP, gnomAD, and an in‐house exome sequencing variant database consisting of 1,092 healthy people. Co‐segregation between the variant and the RP phenotype was confirmed using Sanger sequencing for this family (Figure 1c). Six affected individuals were heterozygous for the variant and two unaffected individuals possessed the wild‐type allele. The novel c.855+5G>A variant in *PRPF31* was completely co‐segregated with the disease phenotype in this family.

In the adRP‐02 family, a novel heterozygous frameshift mutation c.849_855del (p. Pro284Ilefs*35) was identified in the proband (III:5) using WES (Figure 1d). Nine members of the adRP‐02 family (seven unaffected, two affected) were verified to be positive or not for the c.849_855del (p.Pro284Ilefs*35) variant in *PRPF31*, via Sanger sequencing. This variant was not found in dbSNP, gnomAD, and an in‐house exome sequencing variant database consisting of 1,092 healthy people. An affected cousin of the proband (III:3) is a carrier for the c.849_855del (p.Pro284Ilefs*35) variant. This variant is absent in five unaffected subjects. However, two of the asymptomatic individuals, the proband's father and another symptomatic patient’ father, are also carriers for the c.849_855del (p. Pro284Ilefs*35) variant. These results show that the inheritance pattern presents an autosomal dominant inheritance disease with incomplete penetrance.

In the adRP‐01 family, the c.855+5G>A variant in *PRPF31*, located in intronic regions, seemed to involve exonic splicing, according to splicing prediction software. Subsequently, RT‐PCR analysis was performed on *PRPF31* to determine whether the nonclassical splicing mutation has any effect on mRNA splicing. Two PCR fragments of 1.5 kb and 1.1 kb were obtained from the RNA samples extracted from the proband (III:4). A 1.5 kb fragment was amplified from the sample extracted from an unaffected family member (Figure 1e). The results of the Sanger sequencing showed that the 1.5 kb PCR products from the proband (III:4) and unaffected individuals showed the full‐length sequence of *PRPF31* gene (NM_015629.4) cDNA. However, the 1.1 kb fragments from the proband showed a deletion from the 997th to 1405th positions of *PRPF31* gene (NM_015629.4) cDNA, which resulted in alternative splicing of exon 10 to exon 14. The results showed that the *PRPF31* gene c.855+5G>A variant affected the mRNA splicing of the *PRPF31* gene (Figure 1f and g).

### 3.3 Protein structure and Bioinformatics analysis

**FIGURE 2:**
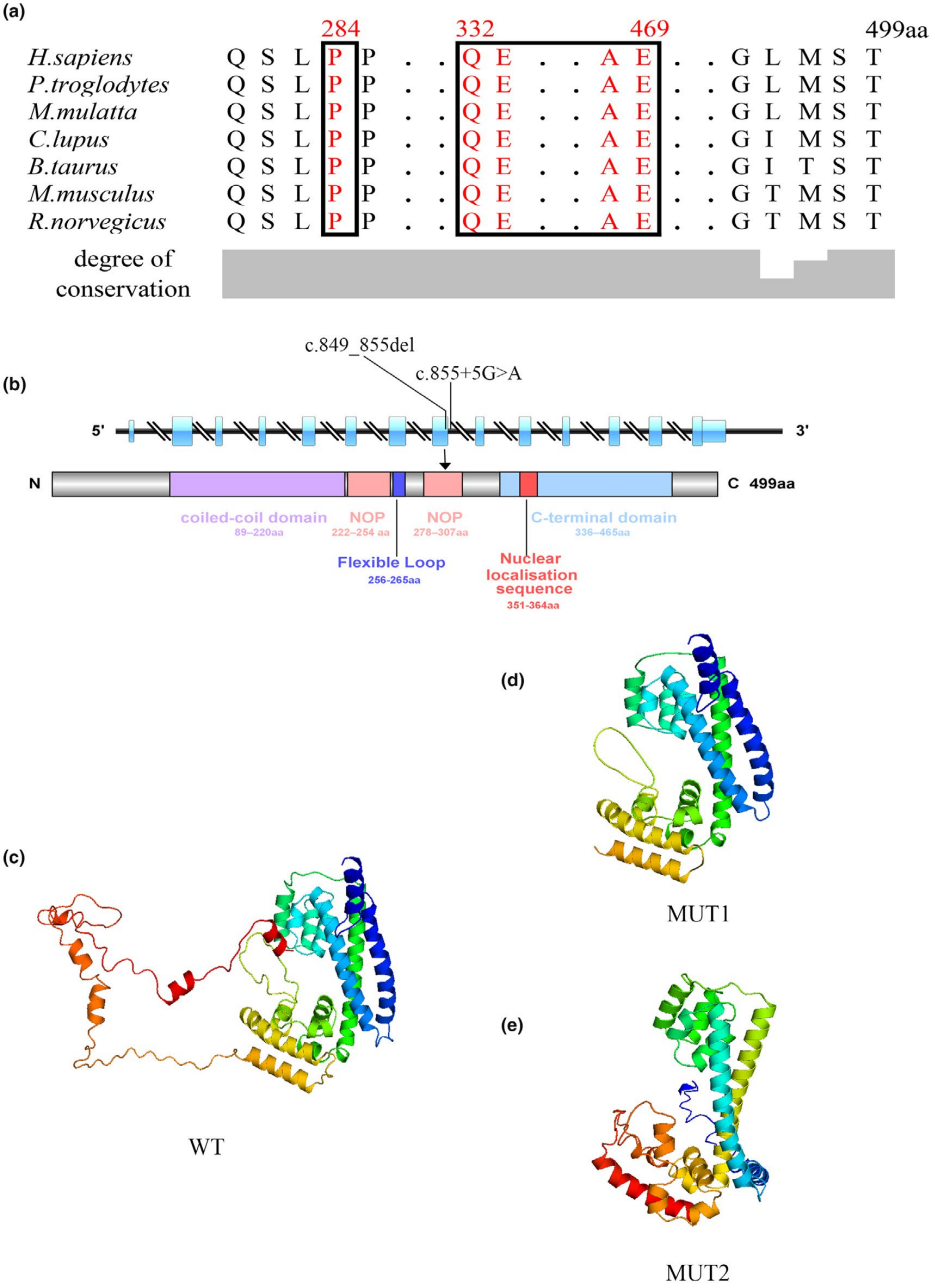
In silico analysis. (a) Evolutionary conservation of the PRPF31 protein (the first 250 amino acids are not shown). The predicted truncated PRPF31 proteins caused by c.855+5G>A and c.849_855del (p.Pro284Ilefs*35) at an evolutionarily conserved amino acid. (b) PRPF31 contains five functional domains. c.855+5G>A and c.849_855del (p.Pro284Ilefs*35) mutations are located in the NOP domain. (c–e) Structure of wild‐type, c.855+5G>A and c.849_855del (p.Pro284Ilefs*35) of PRPF31 by SWISS‐MODEL and shown with Swiss‐PdbViewer. (c) Wild‐type *PRPF31* gene‐encoded protein. (d) MUT1: mutant *PRPF31* (c.855+5G>A mutation) gene‐encoded protein. (e) MUT2: mutant *PRPF31* (c.849_855del mutation) gene‐encoded protein.

The c.855+5G>A variant in *PRPF31* is missing from the 997th to 1405th positions of the *PRPF31* gene (NM_015629.4) cDNA, which changes glutamic acid (acidic amino acid) to arginine aspartic acid (alkaline amino acid) at codon 333. This variant generates a stop codon at position 365, which results in a truncated PRPF31 protein with an aberrant 32‐amino acid residue. The c.849_855del (p.Pro284Ilefs*35) variant is predicted to generate a premature stop codon at codon 318, which putatively generates a truncated PRPF31 protein with 317 amino acids, including an aberrant 34‐amino acid residue. Evolutionary conservation analysis of the amino acid sequences of PRPF31 proteins from different species shows that these impaired PRPF31 regions are highly evolutionary conserved (Figure 2a).

The SWISS‐MODEL analysis demonstrated that the structures of these two mutant proteins lack NOP and C‐terminal domains (Figure 2c).

### 3.4 Functional analysis of the *PRPF31* variant

**FIGURE 3:**
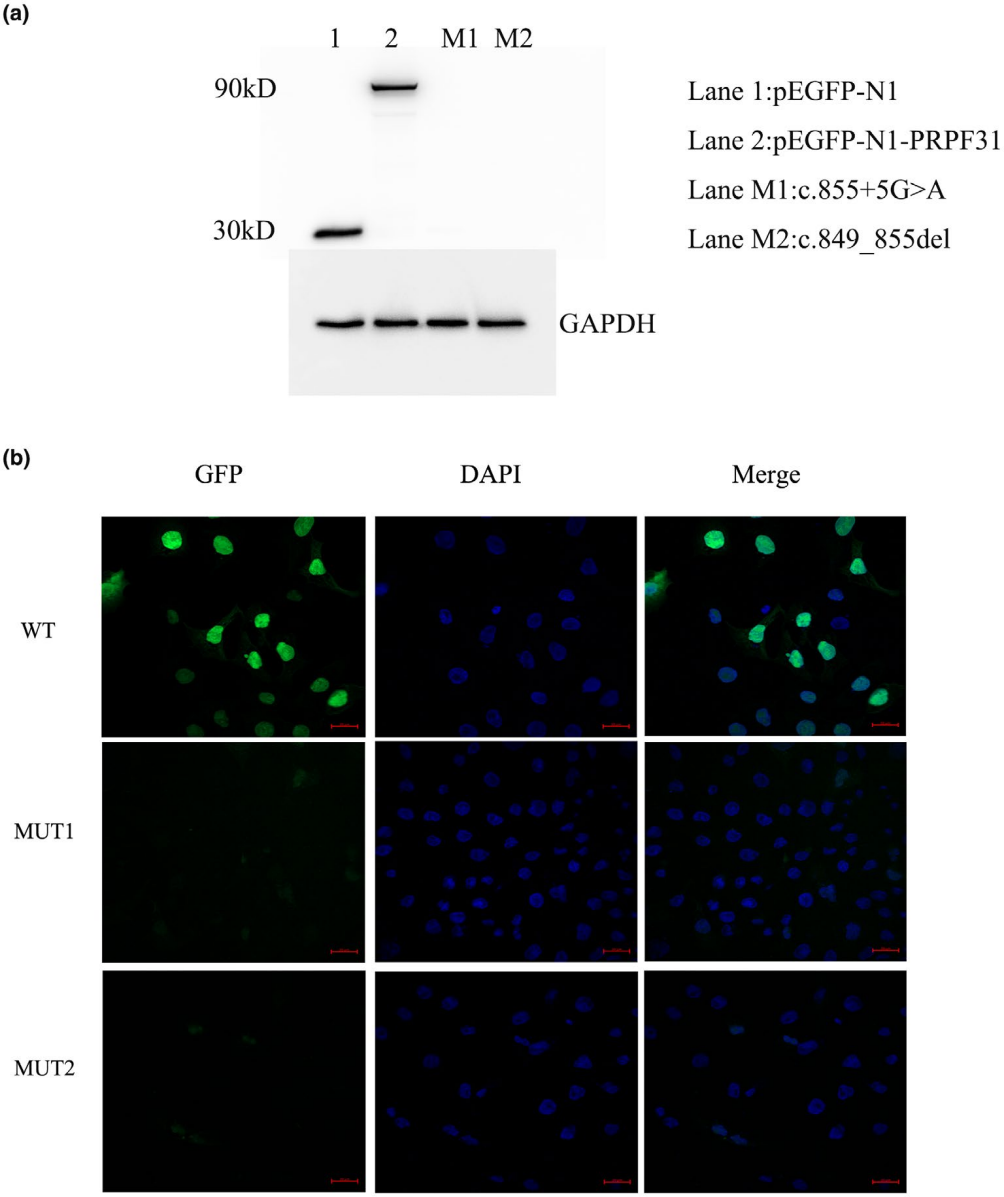
Expression analysis of the two novel *PRPF31* variant. (a)The levels of GFP‐fused PRPF31 were detected using anti‐GFP antibody. From Lane1 to Lane4, respectively, HEK293 cell with transfection of pEGFP‐N1, pEGFP‐N1‐*PRPF31*, pEGFP‐N1‐*PRPF31*M1, or pEGFP‐N1‐*PRPF31*M2. No immunoreactive bands of GFP‐fused PRPF31 mutants are detected by anti‐GFP in 293 T cells. (b) Location of GFP‐fused PRPF31 and the two GFP‐fused PRPF31 mutants in COS7 cells by immunofluorescence for GFP (green). Nuclei show blue due to staining with DAPI. GFP‐fused PRPF31 predominantly located in the nucleus, while the two GFP‐fused PRPF31 mutants were not observed in the COS‐7 cells. Scale bars = 20 µm.

In order to detect whether c.855+ 5G>A and c.849_855del (p.P284Ifs *35) affect the expression of PRPF31 protein in *vitro*, we constructed GFP‐fused *PRPF31* plasmids with a wild type and its mutants. As shown in Figure 3a, the levels of PRPF31 were detected via western blotting of 293 T cells. GFP‐fused PRPF31 of molecular weight 90 kDa were detected in 293 T cells with transfection of the wild‐type GFP‐fused PRPF31 plasmids. No immunoreactive bands of GFP‐fused PRPF31 mutants were detected by anti‐GFP in 293 T cells. We then investigated the expression of PRPF31 in the COS‐7 cells transfected with pEGFP‐N1‐*PRPF31*, pEGFP‐N1‐*PRPF31*M1, or pEGFP‐N1‐*PRPF31*M2 plasmids. Compared with the dispersoid distribution of GFP‐fused PRPF31 in the nucleus, the two GFP‐fused PRPF31 mutants were not observed in the COS‐7 cells, indicating that the variant may not express the GFP‐fused PRPF31 protein (Figure 3b).

## 4 DISCUSSION

Although *PRPF31* is required for splicing in all cell types, rather than only in retinal cells, its pathological effects are observed only in rod photoreceptors. The particular function of PRPF31 in photoreceptor cells remains unknown (Köhn et al., 2009). The PRPF31 protein harbors some functional structural domains: the coiled‐coil domain (89‐220 amino acids), snoRNA binding domain (NOP, 222‐254, and 278‐307 amino acids), flexible loop (256‐265 amino acids), C‐terminal domain (336‐465 amino acids), and nuclear localization sequence (NLS, 351‐364 amino acids; Figure 2b; Wheway et al., 2020). In this study, two novel heterozygous variants of *PRPF31*, c.855+5G>A, and c.849_855del (p. Pro284Ilefs*35) were identified in two Chinese families with adRP.

The c.855+5G>A variant in *PRPF31* was identified as a result of the deletion from the 997th to 1405th positions of the *PRPF31* gene (NM_015629.4) cDNA, which changes glutamic acid (acidic amino acid) to arginine aspartic acid (alkaline amino acid) at codon 333. This variant generates a stop codon at position 365, which results in a truncated PRPF31 protein with the skipping of amino acid 333‐499 amino acid residue. The truncated PRPF31 protein destroys the C‐terminal domain and NLS. NLS domain in PRPF31 protein pilots the location of proteins into the nucleus after translation. There are also several phosphorylation sites on the C‐terminal domain of the PRPF31 protein. A lack of these domains may interfere with the nucleus localization of the PRPF31 protein from the cytoplasm.

The c.849_855del (p.Pro284Ilefs*35) variant is predicted to be responsible for generating a truncated PRPF31 protein with 317 amino acids. No immunoreactive bands of GFP‐fused PRPF31 mutants were detected in 293 T cells with the transfection of GFP‐fused PRPF31 mutant plasmids. Simultaneously, the GFP‐fused PRPF31 mutants were not observed in the COS‐7 cells, indicating that the variant may not express the GFP‐fused PRPF31 protein by producing a truncated PRPF31 protein with 317 amino acids. This truncated PRPF31 protein leads to damage of NOP and a lack of a C‐terminal domain. The NOP domain is an RNP binding module, which exhibits high specificity for binding RNA and protein (Liu et al., 2007). The damage of the NOP domain may interfere with the formation of the spliceosomal complex and eventually affect the splicing process of pre‐mRNA(Liu et al., 2007). Therefore, the splicing mutation (c.855+5G>A) and the deletion‐frameshift mutation (p. Pro284Ilefs*35) in *PRPF31* may interfere with the formation of spliceosomal complex and eventually lead to the development of retinal diseases.

It should be noted that the adRP‐01 family showed a classical autosomal dominant inheritance pattern, whereas asymptomatic gene carriers (II:1 and II:4) were present in the adRP‐02 family. Therefore, we considered the adRP‐02 family to exhibit an incomplete penetrance inheritance. *PRPF31* is most commonly associated with adRP, with incomplete penetrance among seven encoding pre‐mRNA processing factors: *PRPF3*, *PRPF4*, *PRPF6*, *PRPF8*, *PRPF31*, *RP9*, and *SNRNP200* (Yuan et al., 2005). Previously, it was reported that adRP families with *PRPF31* mutations often show incomplete penetrance. Kohn L. claimed that a greater expression of functional *PRPF31* mRNA may protect carriers from the disease and result in incomplete penetrance (Köhn et al., 2009). In other words, incomplete penetrance is due to wild‐type allele overexpression to compensate for nonfunctional alleles in asymptomatic mutation carriers, resulting in normal or mildly affected retinal function being observed in them (Rivolta et al., 2006; Vithana et al., 2003).

In addition to alleles, other genetic factors can also affect the expression level of *PRPF31*. It has been reported that both the minisatellite repeat element (*MSR1*) and cis‐actin transcriptional factor (*CNOT3*) play a significant role in the regulation of *PRPF31* penetrance by modulating its mRNA transcription(Rose et al., 2014, 2016). The *MSR1* element is a 36‐38 bp minisatellite repeat—a cluster of *MSR1* elements located near the *PRPF31* core promoter (~200 bp upstream to the start of the core promoter region). It was demonstrated that three copies of the *MSR1* gene reduced *PRPF31* transcription, while four copies increased *PRPF31* expression, which explained the haploid defects of symptomatic carriers and the normal phenotypes of asymptomatic carriers, respectively (Rose et al., 2016). Copy number variation (CNV) in the *MSR1* elements is a major determinant of *PRPF31*‐induced RP11 disease penetrance. *CNOT3*, a conserved multiprotein structure involved in the regulation of gene expression, belongs to the Ccr4‐Not complex (Collart & Panasenko, 2012). *CNOT3* is a negative regulator that affects the expression of *PRPF31*. In cell experiments, siRNA‐mediated silencing of *CNOT3* resulted in an increase in *PRPF31* expression. When the expression of *CNOT3* is low, *PRPF31* will increase transcription, which can inhibit the expression of disease symptoms in asymptomatic carriers (Venturini et al., 2012). *CNOT3*, similarly to an *MSR1* element, is the main modifier gene determining the penetrance of *PRPF31* mutations.

The penetrance rate of *PRPF31* mutation might be family‐dependent. In our study, pedigree adRP‐01 show a classical autosomal dominant inheritance and adRP‐02 is an incomplete penetrance family. However, especially there are only two unaffected participants in adRP‐01 family. It is necessary that more unaffected individuals from pedigree adRP‐01 are recruited to further determine a relationship between genotypes and phenotypes in the future. In previous report(Xiao et al., 2017), two frameshift variants in PRPF31 were identified in two RP families with complete penetrance and one stopgain variant were identified in a RP family with incomplete penetrance. Compared with the WT, the GFP‐fused PRPF31 protein with two frameshift variants showed a lower level GFP expression. However, the expression of GFP‐PRPF31 containing the stopgain mutation was increased. In our further studies, we will investigate the effect of two variants (c.855+5G>A, c.849_855del) on the expression of PRPF31 protein.

In summary, we found two variants of *PRPF31* in two Chinese families, both of which are predicted to destroy protein function. The c.849_855del (p.Pro284Ilefs*35) and c.855+5G>A variants were categorized as pathogenic variants according to the interpretation guidelines of the ACMG. These variants of *PRPF31* have never been reported before. Our study is of great importance for expanding the knowledge of the significance of the *PRPF31* mutation in RP. Functional analysis has revealed that these variants lead to the truncation of the PRPF31 protein.

## CONFLICT OF INTEREST

No commercial interests exist.

## AUTHORS’ CONTRIBUTIONS

JY.Y. designed the experiments. L.C. and CY.P. collected samples. L.C., CY.P., J.Y., and W.J. performed the experiments. JY.Y., L.C., and CY.P. analyzed the data and drafted the article. JY.Y. revised the article critically for important intellectual content.

## Supporting information

Table S1

## Data Availability

The data that support the findings of this study are available from the corresponding author upon reasonable request.
