# Supplementary material for: Identification of two novel PRPF31 mutations in Chinese families with non‐syndromic autosomal dominant retinitis pigmentosa
Source: Mol Genet Genomic Med. 2020 Oct 21;8(12):e1537. doi: 10.1002/mgg3.1537 (PMC7767543; doi:10.1002/mgg3.1537)
Supplement: Supplementary file 1 — Table S1 [file MGG3-8-e1537-s001.docx]

Supplementary Table S1

Primers sequences used for *PRPF31* gene amplification, sequencing and PCR-based site-directed mutagenesis

| Amplicon | Primers sequence (5’ to 3’) |
| --- | --- |
| c.855+5G>A-F | GACCAACCTCTCCAAGATG |
| c.855+5G>A-R | CATTTGATAGGGGAGGAGAC |
| c.849_855del-F | TTCATGTAAAGGTGCCCAGC |
| c.849_855del-R | TGGCTCTCACGTCCCATG |
| *PRPF31*-cDNA- F | GCCACCATGTCTCTGGCAGATGAGC |
| *PRPF31*-cDNA- R | GGTGGACATAAGGCCACTCTTC |
| pEGFP-N1-F | gaagagtggccttatgtccaccGTGAGCAAGGGCGAGGAGCTG |
| pEGFP-N1-R | gctcatctgccagagacatggtggcGACTGCAGAATTCGAAGCTTG |
| *pEGFP-N1-PRPF31M1-F* | AGAAGAAGGTGGCTGAGG |
| *pEGFP-N1-PRPF31M1-R* | CTGCCACTTGTCGAATTTG |
| *pEGFP-N1-PRPF31M2-F* | CCTGACCAACCTCTCCAAGA |
| *pEGFP-N1-PRPF31M2-R* | GCTCCTTCATCTTGCGGTAC |
